# First-in-human administration of terbium-161-labelled somatostatin receptor subtype 2 antagonist ([^161^Tb]Tb-DOTA-LM3) in a patient with a metastatic neuroendocrine tumour of the ileum

**DOI:** 10.1007/s00259-024-06641-w

**Published:** 2024-03-07

**Authors:** Julia Fricke, Frida Westerbergh, Lisa McDougall, Chiara Favaretto, Emanuel Christ, Guillaume P. Nicolas, Susanne Geistlich, Francesca Borgna, Melpomeni Fani, Peter Bernhardt, Nicholas P. van der Meulen, Cristina Müller, Roger Schibli, Damian Wild

**Affiliations:** 1 Division of Nuclear Medicine, University Hospital Basel, Basel, Switzerland; 2 Department of Medical Radiation Sciences, Institute of Clinical Sciences, Sahlgrenska Academy at University of Gothenburg, Gothenburg, Sweden; 3 Center for Radiopharmaceutical Sciences, Paul Scherrer Institute (PSI), Villigen, Switzerland; 4 ENETS Center of Excellence for Neuroendocrine and Endocrine Tumours, University Hospital Basel, Basel, Switzerland; 5 Division of Endocrinology, University Hospital Basel, Basel, Switzerland; 6 Division of Radiopharmaceutical Chemistry, University Hospital Basel, Basel, Switzerland; 7 Department of Medical Physics and Biomedical Engineering, Sahlgrenska University Hospital, Gothenburg, Sweden; 8 Laboratory of Radiochemistry, Paul Scherrer Institute (PSI), Villigen, Switzerland; 9 Department of Chemistry and Applied Biosciences, ETH Zurich, Zurich, Switzerland

Here, we report on the first patient (78-year-old man) with a metastatic, hormone-active (carcinoid syndrome) ileal neuroendocrine tumour (G1, Ki-67, < 3%), who received a test infusion of 1 GBq [^161^Tb]Tb-DOTA-LM3 in an ongoing prospective Phase 0 study. So far, the patient received long-acting octreotide, which was stopped 2 months before [^161^Tb]Tb-DOTA-LM3 infusion.

Similar to ^177^Lu, ^161^Tb decays with a half-life of 6.95 days and emits medium-energy β^-^-radiation (Eβ_average_ = 154 keV) accompanied by photons suitable for imaging and dosimetry purposes (e.g. Eγ = 49 keV [17%], 75 keV [10%]) [1]. In addition, ^161^Tb also emits conversion electrons and high quantities of Auger electrons (1213%) with a high linear energy transfer over a short distance (< 40 keV/μm). Somatostatin receptor subtype 2 antagonists such as DOTA-LM3 bind to many more binding sites, which leads to a much higher tumour accumulation compared to somatostatin receptor subtype 2 agonists [2]. The preclinical evaluation confirmed the superior therapeutic efficacy of [^161^Tb]Tb-DOTA-LM3 over [^177^Lu]Lu-DOTA-LM3, [^161^Tb]Tb-DOTATOC and [^177^Lu]Lu-DOTATOC, where the latter is routinely used for peptide receptor radionuclide therapy [3].

Maximum intensity projection (MIP) PET image (a) 1 h after i. v. administration of [^68^Ga]Ga-DOTATATE shows moderate tumour burden with several lymph node, liver and peritoneal metastases. MIP SPECT images 24 h (b), 168 h (c) and transaxial SPECT/CT images 168 h (d, e) after infusion of 1 GBq [^161^Tb]Tb-DOTA-LM3 revealed good image quality for both energy windows (75 keV ± 10% and 49 keV ± 20%), despite the low photon energy. Quantitative SPECT/CT imaging was performed 3, 24, 72 and 168 h after infusion of [^161^Tb]Tb-DOTA-LM3 using a LEHR-collimator. Tumour and organ-absorbed doses were calculated using the 75 keV-window and a Monte-Carlo-based OSEM algorithm. The long mean (range) tumour half-life of 130 (123–135) h in liver metastases (red arrows) measuring 3.1–3.3 cm in the contrast-enhanced CT scan (f, g) resulted in mean (range) tumour absorbed dose of 28 (18–39) Gy/GBq. Bone marrow (black triangles), kidney and spleen absorbed dose were determined as 0.31, 3.33 and 6.86 Gy/GBq, respectively. Additionally, a decrease of the tumour marker chromogranin A from 522 to 359 µg/L was measured within 2 months after infusion of only 1 GBq [^161^Tb]Tb-DOTA-LM3. According to CTCAE v5.0, grade 1 thrombocytopenia and grade 3 lymphocytopenia (grade 2 lymphocytopenia was already present at the time of baseline) were observed.

The case presented shows the potential of [^161^Tb]Tb-DOTA-LM3 as a promising alternative to the current standard peptide receptor radionuclide therapy with [^177^Lu]Lu-DOTATOC/[^177^Lu]Lu-DOTATATE (Lutathera®) for patients with metastatic gastroenteropancreatic neuroendocrine tumours.


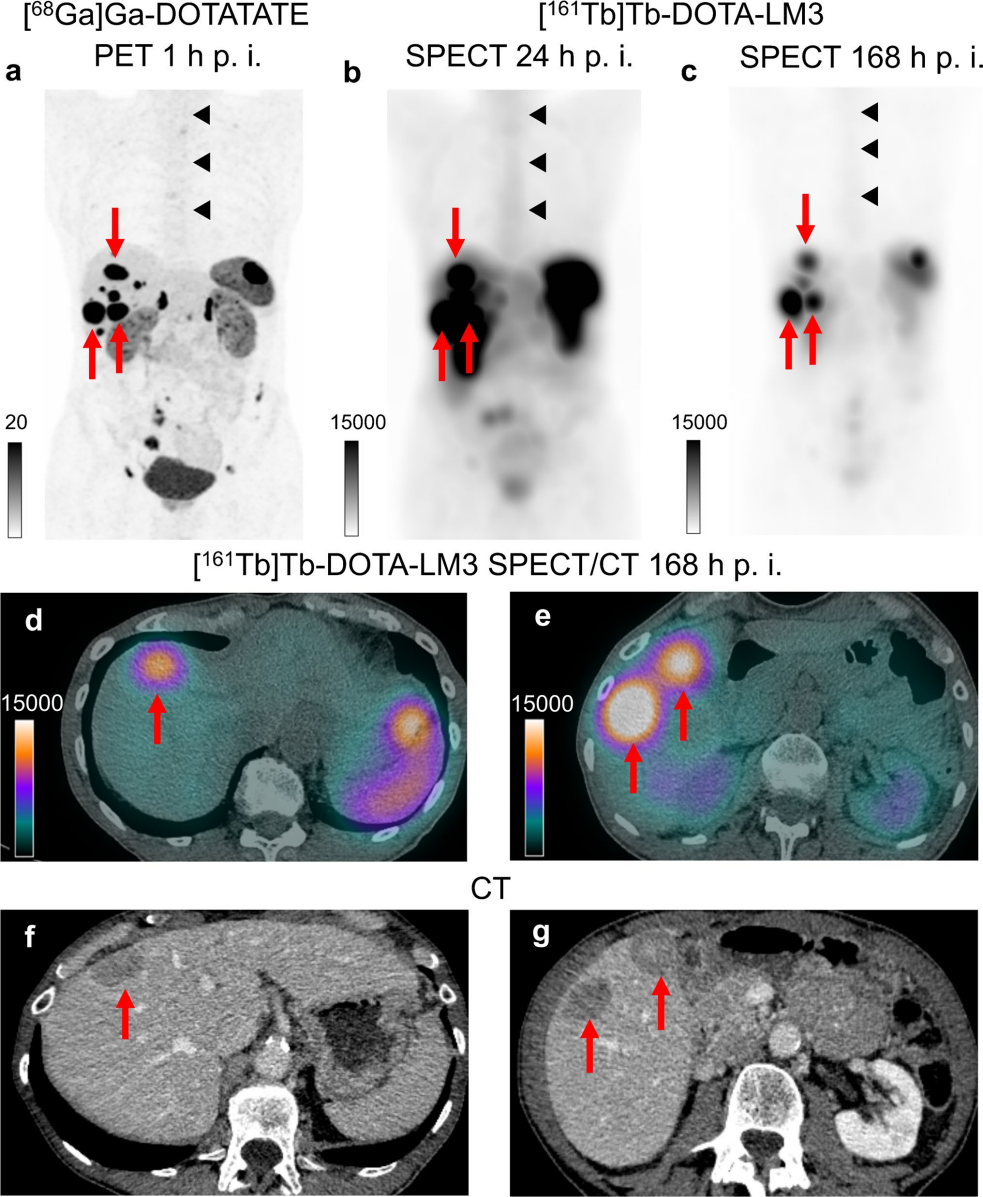


## Data Availability

Data will be made available upon reasonable request.

## Declarations

### Clinical trial registration

This study is registered with ClinicalTrials.gov (NCT05359146).

## References

[1] Marin I, Ryden T, Van Essen M, Svensson J, Gracheva N, Köster U, Zeevaart JR, van der Meulen NP, Müller C, Bernhardt P (2020). Establishment of a clinical SPECT/CT protocol for imaging of ^161^Tb. EJNMMI Phys..

[2] Fani M, Mansi R, Nicolas GP, Wild D (2022). Radiolabeled somatostatin analogs-a continuously evolving class of radiopharmaceuticals. Cancers (Basel).

[3] Borgna F, Haller S, Rodriguez JMM, Ginj M, Grundler PV, Zeevaart JR, Köster U, Schibli R, van der Meulen NP, Müller C. Combination of terbium-161 with somatostatin receptor antagonists-a potential paradigm shift for the treatment of neuroendocrine neoplasms. Eur J Nucl Med Mol Imaging. 2022;49(4):1113-1126. 10.1007/s00259-021-05564-0. 10.1007/s00259-021-05564-0 PMC8921065 34625828

